# Hyperoxaluria leads to dysbiosis and drives selective enrichment of oxalate metabolizing bacterial species in recurrent kidney stone endures

**DOI:** 10.1038/srep34712

**Published:** 2016-10-06

**Authors:** Mangesh V. Suryavanshi, Shrikant S. Bhute, Swapnil D. Jadhav, Manish S. Bhatia, Rahul P. Gune, Yogesh S. Shouche

**Affiliations:** 1 Microbial Culture Collection, National Centre for Cell Science, Central Tower, Sai Trinity Building Garware Circle, Sutarwadi, Pashan Pune 411021 (M.S.), India; 2 Department of Zoology, Savitribai Phule Pune University, Ganesh khind, Pune 411007 (M.S.), India; 3 Department of Pharmaceutical Chemistry, Bharati Vidyapeeth College of Pharmacy, Kolhapur 416013 (M.S.), India; 4 Department of Urology, RCSM Govt. Medical College, CPR Hospital Compound, Bhausingji Rd, Kolhapur 416002 (M.S.), India

## Abstract

Hyperoxaluria due to endogenously synthesized and exogenously ingested oxalates is a leading cause of recurrent oxalate stone formations. Even though, humans largely rely on gut microbiota for oxalate homeostasis, hyperoxaluria associated gut microbiota features remain largely unknown. Based on 16S rRNA gene amplicons, targeted metagenomic sequencing of formyl-CoA transferase (*frc*) gene and qPCR assay, we demonstrate a selective enrichment of Oxalate Metabolizing Bacterial Species (OMBS) in hyperoxaluria condition. Interestingly, higher than usual concentration of oxalate was found inhibitory to many gut microbes, including *Oxalobacter formigenes*, a well-characterized OMBS. In addition a concomitant enrichment of acid tolerant pathobionts in recurrent stone sufferers is observed. Further, specific enzymes participating in oxalate metabolism are found augmented in stone endures. Additionally, hyperoxaluria driven dysbiosis was found to be associated with oxalate content, stone episodes and colonization pattern of *Oxalobacter formigenes*. Thus, we rationalize the first in-depth surveillance of OMBS in the human gut and their association with hyperoxaluria. Our findings can be utilized in the treatment of hyperoxaluria associated recurrent stone episodes.

Oxalate, a metabolic by product excessively found in the systemic fluids of hyperoxaluria patients, has a tendency to crystallize in renal tubules and urine. In due course, it initiates cascades of oxalate salt aggregations and blockages in urinary tract system^1^ and emerges as a principal component of kidney crystals^2^. In hyperoxaluric conditions, oxalate (along with urea and uric acid) acts as anuremic toxin for excretory system^3^, leading to altered renal cell functions^4^ and differential expression of abnormal proteins in nephritic cells^5^. Presence of oxalate kidney stones in humans is therefore regarded as a symptomatic phase of oxalosis and sometimes leads to life threatening patho-physiological conditions like chronic kidney disease^6^. Autosomal recessive genetic defects^7^, excessive dietary intake of oxalates^8^ and lack of oxalate metabolism ability^9^ are some of the possible reasons of hyperoxaluria.

Often, in conditions of elevated oxalates, the gut lumen acts as a primary excretory system to remove excessive oxalates^10^. Once in the gut, oxalates are handled by gut bacteria collectively identified as Oxalate metabolizing Bacterial Species (OMBS) which complement the missing oxalate metabolizing ability in mammalian host (Allison, M. J. and Cook, H. M.^11^). OMBS, through degradation and enhanced excretion, plays an active role in handling and maintaining homeostasis of oxalate in the gut^12^ and also maintains reduced levels of oxalates in systemic fluids^3^. So far, studies concerning hyperoxaluric-gut-microbiome have largely focused on *Oxalobacter formigenes*, a known key player in oxalate homeostasis in the gut. A direct link between lack of colonization of *O. formigenes* as a major risk factor and inverse association with calcium oxalate kidney stones in human^13^ and canines^14^, has been established. The OMBS; including *Oxalobacter formigenes*, share common oxalate-degrading enzymes, including membrane-associated antiporter (*oxIT*), formyl-CoA transferase (*frc*) and oxalyl-CoA decarboxylase (*oxc*)^15^. In all OMBS studies so far, these genes have been detected, and functional genes such as *frc*-gene have been used as molecular marker to assess the active OMBS diversity from soil niche^16^.

In several countries of the world, incidence and frequency of kidney stone disease are higher; these areas are often called stone belt areas^17^. Especially in India, the reason for this high prevalence includes, but is not limited to: large genetic variations, different dietary habits and vast geographic distribution^18^. We noticed that comprehensive reports on oxalate kidney stone-associated dysbiosis in human subjects are lacking. In the present study, using 16S rRNA gene sequencing we assessed differences between the compositions of the gut microbiota in individuals with recurrent kidney stones (hereafter called KSD), which is a symptomatic phase of hyperoxaluria, and healthy individuals (hereafter called HLT). In addition, targeted metagenomic sequencing of formyl-CoA transferase (*frc*) gene and qPCR assays were employed for surveillance of the active OMBS and their association with hyperoxaluria.

## Results

### KSD subjects were hyperoxaluric with multiple CaOx stones

**Figure 1:**
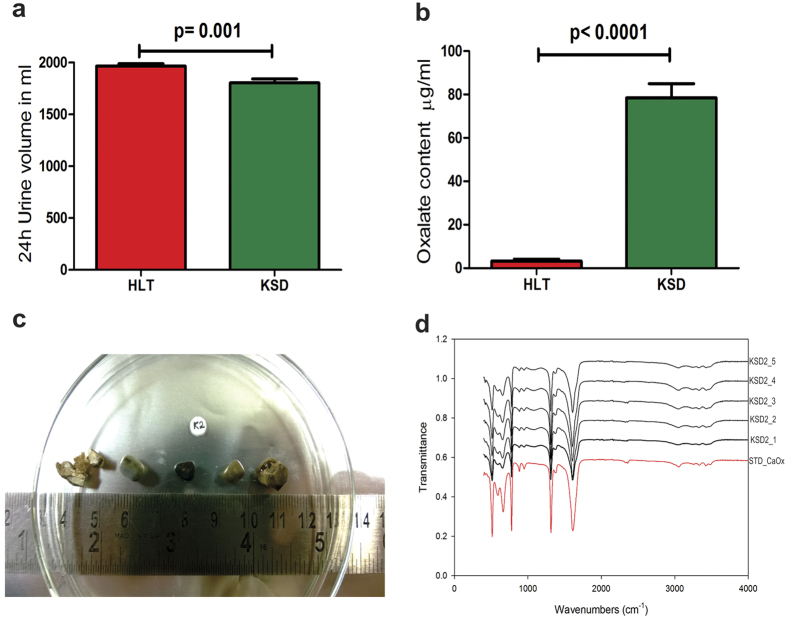
Subjects characterization. (**a**) Total volume measurement 24 h urine data in Mean + SEM. (**b**) Oxalate content in 24 h urine data in Mean + SEM. (**c**) Surgically removed kidney stones from KSD2 subject (representative). (**d**) FTIR spectral evaluation of respective stones from KSD2 subject.

At the time of sampling, KSD subjects (n = 24) were suffering from recurrent kidney stones and were hospitalized for operative procedures; whereas, HLT subjects (n = 15) did not have any apparent complications (Supplementary Table S1). The reduced urine volume in KSD subjects (Mean ± SEM: 1804 ± 38.77 ml) compared to HLT subjects (1967 ± 23.23 ml) was noted (Fig. 1a). Based on oxalate quantification using HPLC method, we demonstrated hyperoxaluric condition in KSD subjects (78.50 ± 6.540 μg/ml) compared to HLT subjects (3.205 ± 0.9099 μg/ml) (Fig. 1b). During the operative procedure, total of 120 stones were recovered from the upper and lower urinary tract of KSD subjects (Fig. 1c), FTIR analysis of these stones revealed that all of them were of calcium oxalate type (Fig. 1d).

### Presence of oxalate kidney stone were associated with dysbiosis in gut microbiota

**Figure 2:**
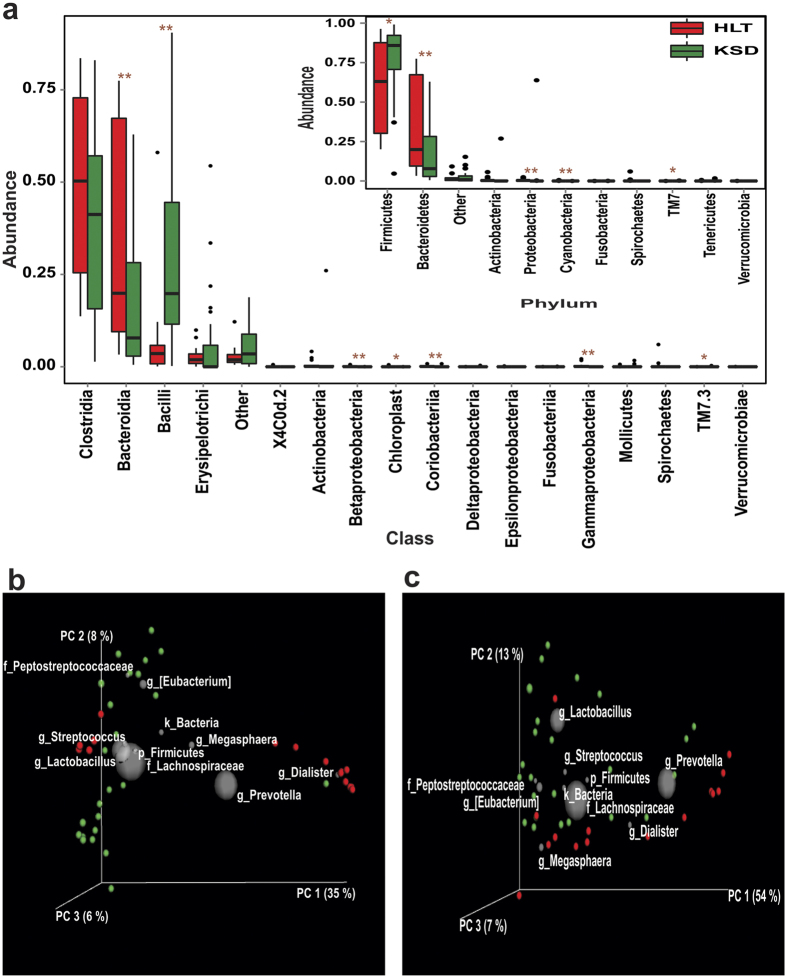
(**a**) Variations in major bacterial phyla and class in HLT and KSD subjects (*p = <0.1, **p = <0.05). PCoA biplot based on (**b**) Unweighted and (**c**) Weighted UniFrac distance matrix: Subjects are represented as HLT (red) and KSD (green) whereas taxonomic group influencing sample segregation are shown as grey sphere whose size demonstrate abundance.

By using Ion Torrent PGM, we obtained ~1.3 million good quality 16S rRNA gene amplicon reads from all the study participants. Using QIIME and UCLUST algorithm, these sequences were grouped into 19,633 unique OTUs. No significant differences were observed in alpha diversity indices viz. Chao1, observed species, phylogenetic diversity, Shannon and Simpson (Supplementary Table S2) between the HLT and KSD subjects. We were able to recover 11 bacterial phyla from all the subjects, when we applied Mann-Whitey U test, notable differences were observed at phylum and class level abundance (Fig. 2a). Particularly, Firmicutes (p = 0.05), Proteobacteria (p = 0.0007) and TM7 (p = 0.044) were found to be increased, while Bacteroidetes (p = 0.007) and Cyanobacteria (p = 0.004) were decreased in KSD subjects. Upon closer examination of taxonomic data, we noted that KSD subjects were enriched with class Bacilli (p = 0.0005), Gammaproteobacteria (p < 0.0001) and TM7-3 (p = 0.044) whereas class Bacteroidia (p = 0.007), Betaproteobacteria (p = 0.0004), Chloroplast (p = 0.013) and Coriobacteriia (p < 0.0001) were reduced in KSD subjects. Beta diversity analysis using unweighted (Fig. 2b) and weighted UniFrac (Fig. 2c) distance matrices revealed compositional differences in overall microbial communities in HLT and KSD subjects. Both these plots suggest that members of Firmicutes such as Lachnospiraceae, Peptostreptocaccaceae, *Streptococcus* and *Lactobacillus* have profound effect on segregation of KSD subjects on PCoA plots.

Kruskal-Wallis (a nonparametric ANOVA) test revealed 1602 significantly differing OTUs (p = <0.01) in HLT and KSD subjects. Of these, 952 were completely absent and 341 were augmented in KSD subjects (Supplementary Table S3). In order to identify strongest taxonomic features responsible for the observed compositional differences in HLT versus KSD subjects we used Random Forest (RF) a supervised-learning model and noticed that there were at least 50 OTUs contributing significantly to the observed differences. Most notably, certain OTUs assigned to *Prevotella* (or *Prevotella copri*) and *Dialister* were solely present in HLT subjects’ whereas OTUs assigned to order Bacteroidales, family Lachnospiraceae and genus *Bacteroides* were dominating in KSD subjects (Supplementary Table S4). Interestingly, *Faecalibacterium prausnitzii* OTUs, one of the dominant butyrate producers in the gut environments was depleted in KSD subjects.

**Figure 3:**
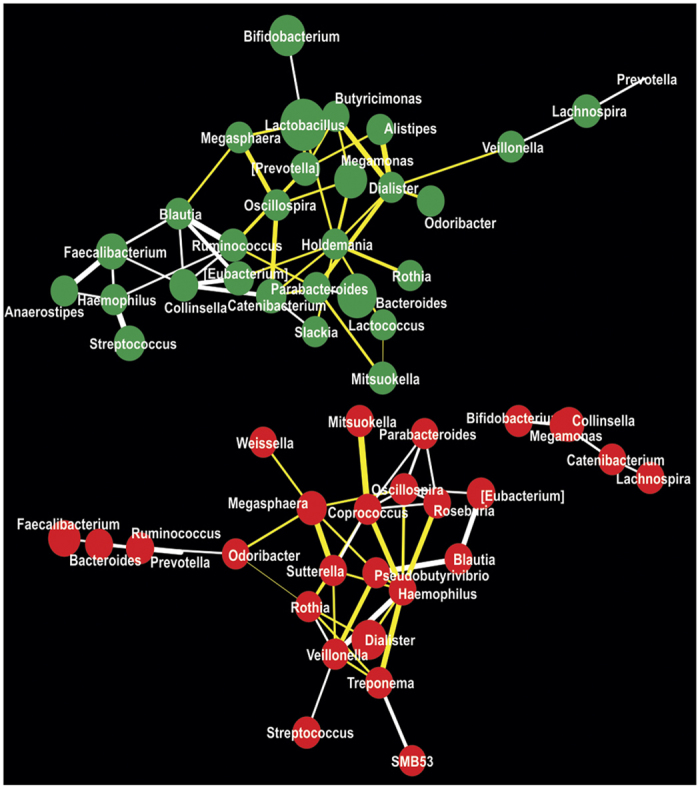
Microbial interaction network in HLT (red colored nodes) and KSD (green colored nodes) subjects represented at genus level. Size of node is indicative of abundance and color of connecting edge indicate interaction type; co-presence: white and co-exclusion: yellow while its thickness represents weight of interactions.

In order to detect altered microbial interactions in the gut due to microbial dysbiosis in KSD subjects, we performed co-occurrence and co-exclusion network analysis (Fig. 3). We used genus level abundance data containing abundance values of 70 detected genera to understand these mutually exclusive interactions. In the resulting network, we were able to detect total of 57 interacting nodes representing 29 (45 interactions) and 28 (44 interactions) significantly interacting genera in KSD and HLT subjects’ respectively. We also noted a concomitant decrease in co-occurrence interactions:23 in HLT versus 18 in KSD and an increase in co-exclusion interactions:21 in HLT versus 27 in KSD. All these observations suggest decrement as well as compositional enrichment in certain bacterial OTUs in KSD subjects as compared to HLT subjects.

### Microbial dysbiosis was associated with the clinical parameters

**Figure 4:**
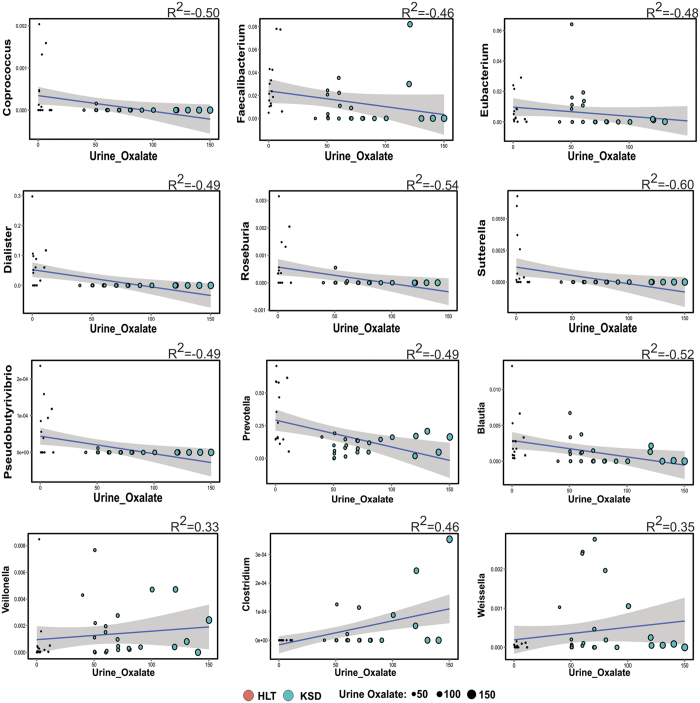
Spearman correlation indicating positive and negative responses of various microbial genera with oxalate concentration in 24 h urine. Linear regression plot displaying best fit blue line with 95% confidence bands and size of sphere corresponds to oxalate concentration.

**Figure 5:**
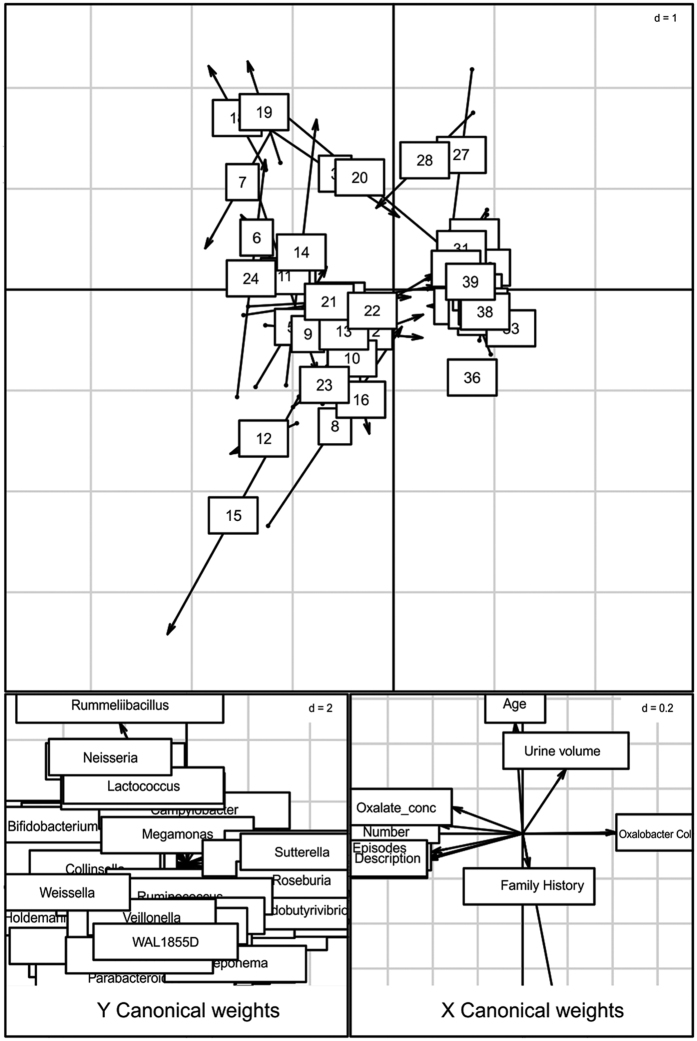
Co-inertia analysis of bacterial genera and subject characteristics. Upper score plot indicate the best matching of 39 subjects with origin of arrows indicating bacterial genera and arrowhead indicating where spots would move relative to subject characteristics. Lower plots shows contribution of two groups of variable to the canonical space.

Simple linear regression analysis revealed that 9 bacterial genera negatively correlated while three positively correlated with the oxalate content (Fig. 4). Most notably, *Prevotella* and *Roseburia*, members of Bacteroidetes phyla and *Faecalibacterium* negatively correlated while *Veillonella, Clostridium* and *Weisella* members of Firmicutes positively correlated with urine oxalate content. We next looked at whether the microbial composition was related to the clinical parameters by performing co-inertia analysis (COIA). COIA (with RV coefficient = 0.307, Monte-Carlo test for 1000 replication p = 0.06) revealed a modest relationship between genus level abundance data and clinical parameters (Fig. 5).

### Imputed metagenome depicted the functional dysbiosis in gut microbial communities of KSD subjects

**Figure 6:**
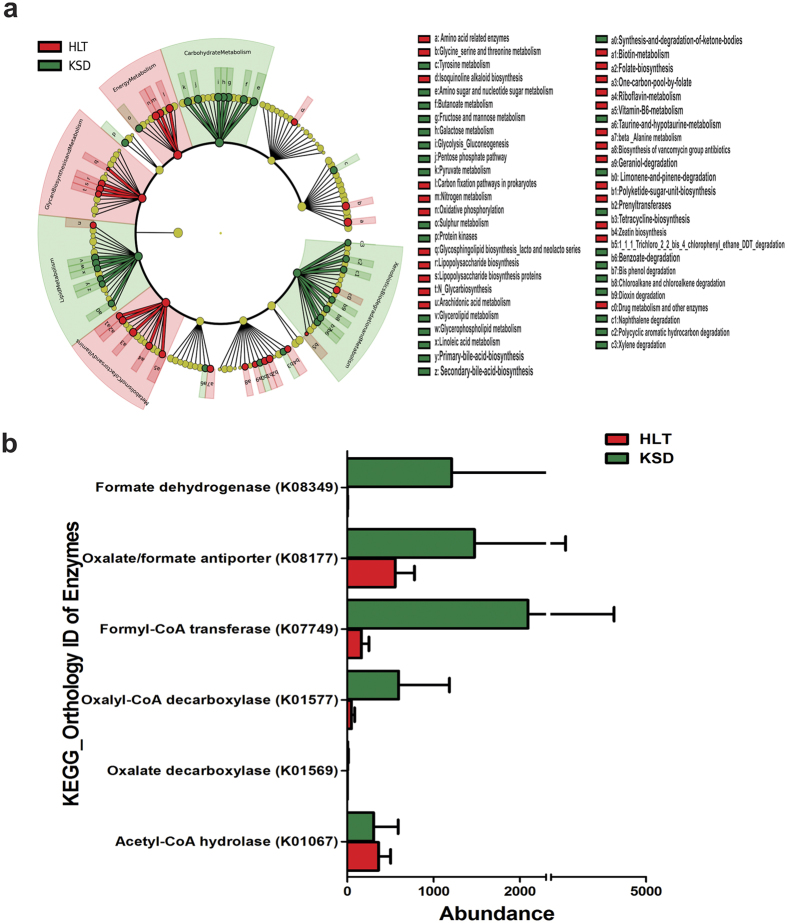
Graphical presentation of imputed metagenome in HLT (red) and KSD (green) subjects. (**a**) Cladogram showing differential abundance of microbial originating metabolic functions. (**b**) Variation in abundance of known KOs involved in oxalate metabolism.

Having observed the microbial dysbiosis in KSD subjects, we were curious to know whether the dysbiosis was associated with specific metabolic enrichments involved in oxalate utilization in these subjects. Hence, metagenomic contributions of gut microbiota were assessed with respect to their ability to utilize oxalate and associated functions using PICRUSt tool. Several gene families were down- regulated or up-regulated in KSD subjects at weighted NSTI (Nearest Sequenced Taxon Index) values of below 0.1. Protein families that were down regulated in KSD subjects were involved in energy metabolism, glycan synthesis and metabolism of co-factors and vitamins. Whereas, protein families up-regulated in KSD subjects include: lipid metabolism, carbohydrate metabolism and xenobiotic degradation and metabolism (Fig. 6a). From KOs, we observed that the enzymes which are involved in oxalate degradation were enriched in KSD subjects, these include formate dehydrogenase (K08349), oxalate/formate antiporter (K08177), formyl-CoA transferase (K07749), oxalyl-CoA decarboxylase (K01577) and oxalate decarboxylase (K01569) (Fig. 6b).

The insignificant difference in abundance of *Oxalobacter formigenes* but the concomitant increase in oxalate metabolizing enzymes KOs in KSD subjects prompted us to find out whether the oxalate metabolism ability is only conferred to well-characterized OMBS in human gut or if there are many other gut residents possessing the ability to utilize oxalate. We therefore segregated the subjects into five groups based on the colonization pattern of *Oxalobacter formigenes*, kidney stone episodes and family history of stone. Colonization of *Oxalobacter formigenes* was confirmed by PCR based approach using specific sets of primers (Supplementary Figure S6). Accordingly, all HLT group subjects and only 04 KSD subjects were found colonized with *Oxalobacter formigenes*, hence five groups consisting of four subjects in each were formed (Table 1). We performed the remaining analysis of these groups which included identification of shared OTUs among the groups and diversity of OMBS by using *frc*-gene as a molecular marker.

### *frc*-gene amplicon sequencing revealed selective enrichment of oxalate metabolizing bacterial communities

**Figure 7:**
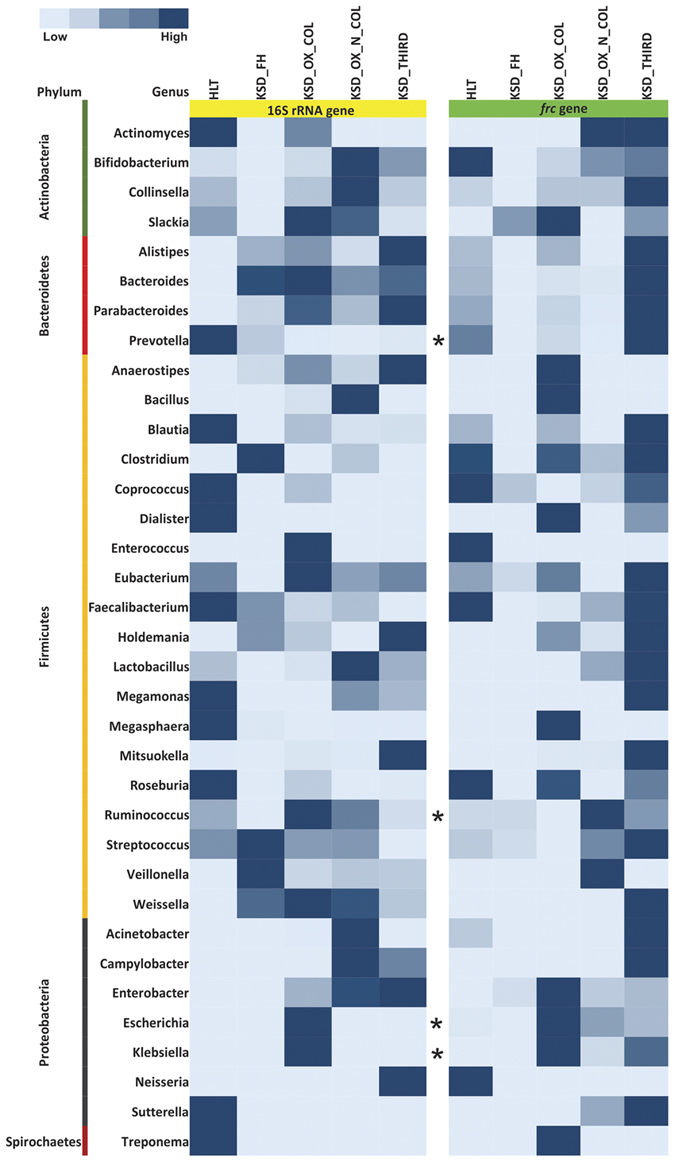
Heatmap representing common bacterial genera detected through 16S rRNA and *frc-*gene amplicon libraries in 5 groups. *Indicates presence of bacterial genera in PCR-DGGE profile.

We first performed DGGE fingerprinting on a subset of samples for *frc*-gene and the differential DGGE pattern was observed (Supplementary Figure S7). Among the bands eluted and sequenced, many sequences had mixed peaks suggesting that these bands are indeed a mixture of two or more DNA sequences. To confirm above results, we selectively amplified the *frc*-gene from metagenomic DNA from all the subjects. Resulting metagenomic data was analysed using MG-RAST server to obtain taxonomic assignments using M5NR database. This way, we were able to identify 289 differentially abundant gut inhabitants which possessed *frc*-gene (Supplementary Table S5) among the different KSD groups and HLT subjects. Of these, 29 were consistently present in all the subjects. Most notable of these were *Oxalobacter formigenes, Methylobacterium populi, Janthinobacterium sp. Marseille, Escherichia coli, Shigella dysenteriae, Oligotropha carboxidovorans, Kribbella flavida,* and *Herminiimonas arsenicoxydans*. Surprisingly, among the 289 bacterial genera 172 were absent in HLT subjects. This indicates that the overall diversity of OMBS in human gut under the selective pressure of hyperoxaluria rises over several orders of magnitude in KSD subjects leading to specific enrichment of OMBS in them. We next extracted common bacterial members which we could observe in both 16S rRNA gene sequence data and *frc*-gene amplicon data, and obtained 35 common bacterial genera. The relative abundance of most of these 35 genera was higher in four KSD groups compared to HLT subjects (Fig. 7), further confirming the enrichment of these genera in KSD subjects.

**Figure 8:**
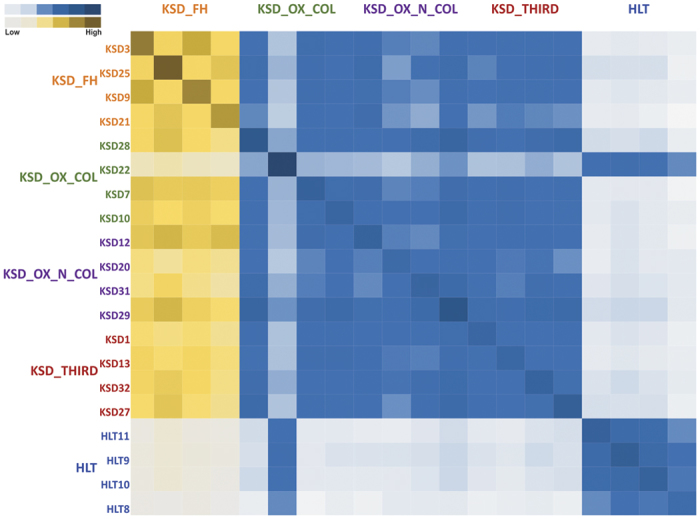
Heatmap representing pairwise inter-individual sharing of phylotypes amongst 5 groups. Gradation of yellow color indicates sharing of phylotypes within KSD groups and blue color indicates sharing of phylotypes between HLT and KSD groups.

In addition, we also performed an analysis of shared phylotypes among the KSD groups and HLT subjects. We found that all members of the individual KSD groups shared maximum phylotypes, while there was gradation in the shared phylotype pattern among the different KSD groups. All the members of all KSD groups (except KSD 22 sample) shared least phylotypes with the members of HLT group which in turn, shared maximum of phylotypes among themselves (Fig. 8). From the cumulative surveillance using 16S rRNA and *frc*-gene amplicon we report dysbiosis and differential enrichment of specific phylotypes in KSD subjects.

### qPCR based assays confirms the enrichment of OMBS in hyperoxaluric condition

**Figure 9:**
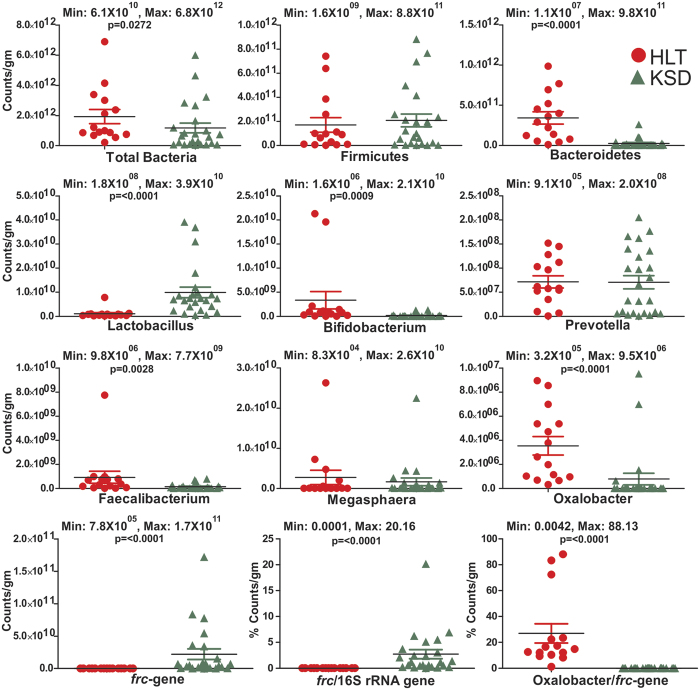
Dot plots representing the counts of selected bacterial taxa and genes by qPCR assay in HLT and KSD subjects. Minimum and maximum count values are displayed for each plot in all tested subjects.

By using qPCR study, we were able to detect and support the amplicon generated results from the quantitative measurements of selected bacteria. Substantial enrichment of *Lactobacillus* group (p = <0.0001) and *frc*-gene (p = <0.0001); whereas depletion in Bacteriodetes phylum (p = <0.0001), *Bifidobacterium* (p = 0.0009), *Fecalibacterium* (p = 0.0028) and *Oxalobacter formigenes* (p = <0.0001) microbial taxa were detected in KSD subjects (Fig. 9). Quantitative PCR results revealed that known OMBS *Oxalobacter formigenes* varied from 3.2 × 10^5^ to 8.9 × 10^6^ counts per gram of fecal sample in HLT subjects and that it was significantly higher. The *frc-*gene is referred to as molecular marker for oxalate metabolizing bacteria and its higher copies can be considered as a signal for enrichment of range of OMBS bacteria in the human gut. The *frc-*gene copies in HLT subjects ranged from 7.8 × 10^5^ to 7.7 × 10^7^ counts per gram which was significantly lower than its copies in KSD subjects 7.4 × 10^7^ to 1.7 × 10^11^. Further, we obtained the overall abundance of OMBS bacteria by taking the ratio of *frc*-gene copy number to 16S rRNA gene copy number and observed that the ratio was much higher for KSD subjects (range: 0.03 to 20.1%) than in HLT subjects (range: 0.00018 to 0.01%). This confirmed the fact that in diseased condition active OMBS are highly enriched. We next obtained the contribution of *Oxalobacter formigenes* to total OMBS population with the help of ratio of *Oxalobacter* copy number to *frc*-gene copy number. Higher ratio of *Oxalobacter formigenes* in HLT subjects (range: 1.2 to 88.13%) indicates that it is an important contributor for oxalate homeostasis in the gut of HLT subjects. However, significantly low ratio in KSD subjects (range: 0.004 to 0.22%) indicate its inhibition and associated enrichment of other OMBS in KSD subjects.

## Discussion

Kidney stone is believed to be a multifactorial disease influenced by lifestyle as well as food habits. In general population, calcium oxalate kidney stones are the most common type of kidney stones and are predominantly found in males than in females^19^. Although, kidney stone is perceived as an acute disease, evidence suggests the fact that for many individuals, hyperoxaluria is a chronic condition and often leads to recurrent stone episodes^1^. In the last two decades, major efforts have been taken in diagnosis and treatment of recurrent kidney stones, however, satisfactory regimes are still awaited. There are a few reasons for unsuccessful treatments including: insufficient information on oxalate content in food, relation between dietary-oxalate precursors and oxalate excretion, and the factors involved in intestinal oxalate handling^20^. Uremic toxin including oxalate may alter the gut biochemical milieu, which may consequently affect structure and composition of gut microbial communities^3^. Previous observations confirm altered microflora with respect to chronic kidney disease^21^,^22^, but specific reports on alteration in gut microbiome associated with oxalate stones in human subjects are scanty^23^. In the present study, we reveal differences in bacterial community structure in 24 male subjects suffering from idiopathic hyperoxaluria and show enrichment of oxalate metabolizing microbes in their gut.

Our results, both at broader and refined levels of microbial taxonomy indicate dysbiosis in major gut microbial communities in these subjects and are in accordance with the previous study in canines^24^. Members of two bacterial phyla Firmicutes and Bacteroidetes dominate endogenous gut microflora of many mammals including humans, forming complex interactions among themselves and with the host to ensure the stability in this ecosystem. Increased abundance of Firmicutes has been linked with metabolic disorders such as obesity^25^ and diabetes^26^, thus augmentation of Firmicutes in hyperoxaluria can be easily perceived. Bacteroidetes are often involved in metabolism of complex polysaccharides mainly derived from the food^26^. Recently, it has been shown that this rather copious phyla possesses contact-dependent inter-bacterial antagonism which is essential to maintain stability of gut microbiota in healthy subjects and also presents a barrier to most of the pathogens like members of Proteobacteria^27^. Indeed, significant decrease in Bacteroidetes and concomitant increase in Proteobacteria in KSD subjects indicates demolition of Bacteroidetes driven inter-bacterial antagonism. Using beta diversity analysis based on unweighted and weighted UniFrac distance matrix, we further confirmed dominance of Firmicutes and deprivation of Bacteroidetes in KSD subjects leading to their distinct segregation on PCoA plots from HLT subjects. Furthermore, dispersal of KSD subjects on PCoA plots indicates enhanced beta diversity associated with enrichment of specific microbial communities in them compared to the HLT subjects. Random Forest machine learning approach is an effective way to identify discriminating taxa between different physiological states or disease conditions^28^. Our observation of higher number of *Prevotella* and Lachnospiraceae OTUs in HLT and KSD subjects respectively, further supports the fact KSD subjects were depleted with Bacteroidetes. Our findings of decreased abundance of *Prevotella* and *Eubacterium* in KSD subjects arein congruent with recent study reporting the variation in gut microbial communities of stone formers and non-stone formers^22^. This same study reports the increased abundance of Bacteroidetes, which is not seen in our study. The observed differences could be attributed to recruitment of more female subjects, stone formers with mixed types of stones and stone formers with other disease-associated complications in the Stern *et al*. study^22^.

Competitive and cooperative interactions are most common microbial interactions occurring in various ecosystems. To obtain complete overview of microbial interactions in human gut is rather difficult task primarily due to the complex nature of this ecosystem and partly due to the fact that many members are yet to be cultured. Hence, few indirect methods have been developed to model these interactions using microbial abundance data^29^. Accordingly, increased competitive interactions in KSD subjects could be related to dysbiosis of healthy microflora in them. Such competitive interactions have been linked with evolution of cooperation in yeast communities^30^.

Association of inverse relationship between *Oxalobacter formigenes* with incidence of kidney stones is well established^12^,^13^. Several studies are describing *Oxalobacter formigenes* as potential probiotics in treatment of hyperoxaluria^31^,^32^; in fact several commercial probiotics products based on O*xalobacter formigenes* are already available in the market^33^. One striking observation of our study is that the taxonomic assignments to the *Oxalobacter formigenes* OTUs could maximally be observed till family level and that this does not differ significantly between HLT and KSD subjects. This could partly be attributed to low abundance of this bacterium in the gut environment as well as its detection sensitivity using V1–V3 region of 16S rRNA gene amplicon sequencing^34^. This was further confirmed by using *Oxalobacter* specific 16S rRNA gene primers which demonstrated its presence in 100% (15 out of 15) HLT subjects as against ~17% (4 out of 24) KSD subjects. Furthermore, our observation of minimum sharing of phylotypes between different KSD groups and HLT subjects, and their gradation pattern in KSD subject, signifies the role of hyperoxaluria in dysbiosis and may be attributed to disease state, family history, stone episodes and presence of *Oxalobacter formigenes*.

We also observed that some of the prominent gut residents to be negatively correlated with the urinary oxalate, even though they have ability to metabolize oxalate. This indicates that above certain concentration, oxalate could be toxic to these common gut inhabitants; similar results have also been observed with respect to *Oxalobacter formigenes*^14^. The co-inertia analys is used to examine global similarity between clinical parameters and genus abundance profile also revealed segregation of *Prevotella, Sutterella, Roseburia* (negatively correlated genera) from *Veillonella* and *Weissella* (positively correlated genera) and we believe that this could be influenced by oxalate concentration, stone number and stone episodes.

Although PICRUSt infer metabolism of given microbiome based on 16S rRNA amplicon sequences, lower NSTI scores are often indicative of good metagenomic predictions. Using KEGG (Kyoto Encyclopedia of Genes and Genomes)^35^, we identified 5 metabolic pathways involved in oxalate degradation in microbes. Enrichment of some of these KOs in KSD subjects further strengthen the observation that microbiota not only alters structurally, but also leads to enrichment of oxalate metabolic function due to hyperoxaluria driven selective pressure. While, our PICRUSt analysis revealed several other important differences in the inferred metagenomic data in relation to calcium oxalate stone formation on a broader scale (Fig. 5a); their augmentation or diminution in KSD subjects needs experimental validation.

Bacterial communities are often tested for their ability to metabolize oxalate by molecular characterization of *frc*-gene. Such studies have highlighted the fact that soils enriched with oxalate, bear highest load of microbes involved in oxalate degradation^15^. In another study, presence and distribution of oxalate utilizing bacterial consortia have been demonstrated throughout the gut ecosystem of herbivorous mammals^36^. To this end we applied DGGE fingerprinting and *frc*-gene amplicon sequence analysis, and for the first time, report the diversity of *frc*-gene in human gut microbial communities in the context of their abilities to metabolize oxalate. Significant finding from this part of our study is that in addition to *Oxalobacter formigenes*, several gut inhabitants possess the *frc*-gene and hence the ability to utilize oxalate. This becomes especially important because humans do not have ability to metabolize oxalates, and are dependent on gut microbial reservoir for oxalate clearance from the gut environment. Furthermore, our results show increased diversity of *frc*-gene in KSD subject indicating enrichment of particular microbial communities involved in utilization of oxalates; this could be a consequence of hyperoxaluria which is not observed in healthy subjects. Since, gut-inhabiting *E. coli* uses homolog of *frc* and *oxc*-gene viz. *YfdW* and *YfdU* for acid tolerance^37^, we further hypothesize that this increased diversity of *frc*-gene could be linked with acquisition of oxalate-induced acid tolerance phenotype, and it may also be due to the inter-species horizontal gene transfer. Conflicting with earlier studies, the presence of *frc*-gene is limited to Actinobacteria, Firmicutes and Proteobacteria phyla^38^. We were able to detect its presence in Bacteroidetes as well as Spirochaetes phyla suggesting that catalogue of *frc*-gene is incomplete and we speculate that it could be extended in many other bacterial phyla.

Absolute quantification of specific bacterial groups in stool samples have been precisely determined by using real-time PCR (qPCR) methods^39^,^40^. qPCR has also been used as a method to validate the findings of next-generation sequencing and micro-RNA data^41^. Hence, we applied qPCR to confirm the major findings of our NGS data. In our opinion this is the first study to report the *frc*-gene frequency in the human gut and its profound association with the oxalate stones. Further, high abundance of *Oxalobacter formigenes* in HLT subjects, low abundance in KSD subjects and its minimal contribution to oxalate metabolism in KSD subjects suggests that in the hyperoxaluria condition, the gut milieu may become unfavorable for its growth. It should also be noted that the *frc* is just one route involved in oxalate metabolism, as per our PICRUSt analysis using KEGG server there are at least five different oxalate degrading pathways, hence, the possibility of existence of alternative pathways of oxalate metabolism should not be neglected^11^,^36^,^42^. However to prove the existence of such alternative pathways there is a need of large scale metagenomic studies.

Some strong aspects of our study are the inclusion of subjects of matching age, who followed vegetarian diet and were from similar socioeconomic class. Exclusion of female subjects and the cross-sectional nature of the study could be the limitations of our study. Further, our findings are largely based on culture-independent studies, considering the huge number of positively associated microbial taxa with oxalate content, there is great scope of culture-dependent studies to understand the contribution of individual microbial taxa in oxalate homeostasis in the human gut. Moreover, since the diet is known to alter the gut microbiota^26^, it is essential to evaluate the effect of specific diet (e.g. oxalate rich food) and associated microbiota towards the development of stones.

In conclusion, our study using the high throughput DNA sequencing reports the dysbiosis of gut microbial communities in recurrent oxalate kidney stone sufferers. Also, it highlights augmentation of structural and functional diversity of oxalate metabolizing bacteria in stone endures. Considering the incidence rate^17^ and recommended therapy for removal of kidney stones^43^, our study provides important avenue in the form of gut microbiota as a potential target for treatment and/or controlling the recurrent episodes of oxalate associated stones.

## Methods

### Participants and Sample Collection

National Centre for Cell Science’s institutional ethics committee approved case-control study. In accordance with declaration of Helsinki principles; a total of thirty nine subjects, symptomatic kidney stone diseased (n = 24) hereafter referred to as KSD, and Healthy control (n = 15) hereafter will be called HLT were involved in the present study. All the subjects were enrolled with their separate written informed consent for this study. Patients who have confirmed nephrolithisis by ultra-imagine; have recurrent stone episodes and have not received any antibiotics or probiotic preparations for last three months before enrolment were included. 24 h urine, surgically removed kidney stones and fecal samples from each KSD group and 24 h urine and fecal samples from each HLT group were collected. Fecal samples from the above subjects were collected in sterile container and stored at −80 °C until they were used for DNA extraction. For subject characterization, 24 h urine and surgically removed kidney stone samples were utilized for oxalate quantification and chemical analysis respectively; while the fecal samples were used for bacterial diversity and targeted metagenomic analysis.

### Subject characterization

In order to define hyperoxaluric condition in KSD subjects, oxalate quantification from 24 h urine sample and analysis of chemical composition of stone were performed. For oxalate quantification, modified HPLC-based method^44^ was developed (detailed in Supplementary Methods S8). Chemical analysis of kidney stones was performed using FTIR analysis. Briefly, one hundred and twenty surgically removed kidney stones were collected in sterile containers; pulverized into powdered form, part of which was analysed by FTIR spectroscopy without KBr method to identify the stone type. By using Bruker FTIR ATR Tensor 37 spectrometer, each stone spectra were obtained in transmittance mode from 4000 to 400 cm^−1^; 32 scans were averaged with a 4 cm^−1^ resolution. Chemical composition determined from resulted spectra for all 120 stones were compared to spectrum of standard CaOx (Sigma Aldrich).

### DNA extraction from fecal samples

Total community DNA was extracted from each faecal samples using QIAmp DNA Stool Mini kit (Qiagen, Madison USA) as per manufacturer’s protocol. The concentration of resulting DNA was measured using Nanodrop-1000, (Thermo Scientific, USA). DNA concentration was normalised to 100 ng/μl and used as template for amplification bacterial genes.

### 16S rRNA gene amplicon sequencing and bioinformatics analysis

16S rRNA gene was amplified using AmpliTaq Gold PCR 360 Master Mix (Life Technologies, USA) and with V3 region specific bacterial universal primers reported earlier^45^. Sequencing and bioinformatics analysis was performed as described previously^46^. Briefly, library generations, production of Ion sphere particles and sequencing of libraries were performed on 316 chips using Ion Torrent PGM system as per manufacturer’s instructions. The raw reads were subjected to quality filtering using MOTHUR pipeline^47^, good quality reads were analysed using QIIME^48^. OTU picking using Greengenes database 13.8^49^ at 97% similarity was performed using open OTU picking approach and representative sequence from each OTU was used to generate OTU table, which was used for subsequent analysis. This includes alpha diversity indices, beta diversity analysis using UniFrac and Principle Coordinate Analysis (PCoA). We next utilize Random Forest approach to identify OTUs that were indicators of community differences. This was done by estimating the amount of error introduced if a particular OTU is removed from a group of indicator OTUs and assigning it an importance score. We considered only those OTUs as highly discriminative if its mean decrease in accuracy was greater than 0.001. Raw sequences generated in the present study are deposited to NCBI Sequence Read Archive under accession number **SRP066940**.

### Subject grouping based on stone characteristics and presence of *Oxalobacter formigenes*

We next divided the study participants based on family history, frequency of stone formation and colonization status of *Oxalobacter formigenes*. The rationale of subject segregation especially of KSD subjects was substantially higher risk of stone formation in male patients having family history^50^. Further, the extreme case of stone endures within the study participants (third time episode) considering the fact that higher the number of stone episodes, more are the chances of development of chronic kidney diseases^51^ and the inverse relationship of *Oxalobacter formigenes* with the stone formation^14^. To show the presence of *Oxalobacter formigenes*, PCR based detection using genus specific primers for 16S rRNA gene and Oxalyl CoA Decarboxylase (*oxc*) gene was performed^52^. Thus, the subjects were grouped into five categories with four subjects in each group viz. KSD with family history (KSD_FH), KSD *O. formigenes* colonizer (KSD_OX_COL), KSD *O. formigenes* non-colonizer (KSD_OX_N_COL), KSD with third episode (KSD_THIRD) and Healthy control (HLT).

### Targeted Functional gene analysis

Owing to the fact that the oxalate metabolism phenomenon is principally observed in microbes, we assessed the gut microbial communities for the presence of formyl CoA transferase gene (*frc*). We used DGGE and targeted metagenomic sequencing to characterize the *frc*- gene as described below:

### DGGE based *frc*-gene diversity analysis

DGGE fingerprinting strategy was used to obtain community structure and to identify oxalotrophic bacterial players by using *frc*-gene specific modified linker primers *frc*171-F (5′-CTSTAYTTCACSATGCTSAAC-3′) and GC-*frc*627-R (5′-TGCTGRTCRCGYAGYTTSAC-3′) as described earlier^15^. PCR was set up in 50 μl reaction using AmpliTaq Gold PCR Master Mix (Life Technologies, USA) and with following conditions were used for touch-down PCR: initial denaturation at 95 °C for 10 min, followed by 10 cycles with reduction of 1 °C in annealing temperature as of 95 °C for 1 min, 56 °C for 30 s, and 72 °C for 30 s and next by 32 cycles of 95 °C for 1 min, 56 °C for 30 s, and 72 °C for 30 s with final extension at 72 °C for 10 min. The PCR products were purified in 15 microliter by sodium acetate precipitation method. Total volume products were then subjected to DGGE in 10% acrylamide: bis acrylamide (37.5:1) gel with a gradient of 25% to 65% of denaturants. The electrophoresis was performed using DCode Universal Mutation Detection System (BioRad, Hercules, CA, USA) in 1 X TAE buffer (40 mM Tris, 20 mM Sodium acetate, 1 mM EDTA) at 80 V and 60 °C for 18 h. The gel was stained with SYBR Gold (Invitrogen) for 20 minutes, visualized using SynGene G: box gel documentation system and analyzed using GeneTools software packages (SynGene, Cambridge, UK). Separated bands were excised, allowed to diffuse in 10 μl distilled water at 37 °C overnight and subjected to sequencing using the same primer set. The sequencing was performed on ABI 3730 XL DNA analyser (Applied Biosystems Inc, USA) using the ABI Big-Dye terminator version 3.1 sequencing kit as per the manufacturer’s instructions.

Generated sequences were BLAST analyzed over NCBI database and bacterial identity hits were recorded.

### *frc*-gene amplicon generation and sequencing

The functional *frc*-gene was PCR amplified using primers *frc*171-F (5′-CTSTAYTTCACSATGCTSAAC-3′) and *frc*306-R (5′-GDSAAGCCCATVCGRTC-3′) as described earlier^15^. The resulting PCR products were purified using Agencourt AMPure XP DNA purification Bead (Beckman Coulter, USA) and quantified using Nanodrop-1000 (Thermo Scientific, USA). Then, PCR products were pooled by mixing equal quantities of concentration normalized PCR products in five group pools. All the pooled group samples were then sequenced as described above.

### *frc*-gene sequence analysis

The fastq sequence files for the *frc*-gene amplicon were uploaded and analyzed using the MetaGenome Rapid Annotation with Subsystem Technology (MG-RAST) server, version 3.6^53^. The raw reads underwent the quality filtering steps and host specific reads were excluded. Phylogenetic analysis was performed using default setting as detailed in MG-RAST manual version 3.6 revision 3 (ftp://ftp.metagenomics.anl.gov/data/manual/mg-rast-manual.pdf). Organism abundance up to species level was obtained by using phylogenetic analysis with Best Hit Classification approach and M5NR database with minimum e-value and identity of 1e-5 and 80% respectively.

### Absolute quantification of specific bacterial taxa and *frc*-gene

In order to confirm the increased or decreased abundance of specific bacterial taxa in HLT and KSD subjects, absolute quantification of 16S rRNA gene copy numbers were estimated using qPCR assay. Additionally, to confirm the enrichment of OMBS in KSD subjects absolute quantification of *frc* and *oxc* genes were performed. Targeted groups of bacteria, primer sequences, and amplicon size are summarized in Table 2. Absolute quantification qPCR assays were performed as described earlier^54^. Briefly, for each gene under consideration triplicate qPCR reactions were setup (10 μl each) containing appropriate pair of primers, 50 μg of metagenomic DNA and SYBR green master mix (Applied biosystems Inc. USA). The reactions were run on 7300 Real time PCR system from Applied Biosystems Inc. (USA) using following PCR conditions: initial denaturation at 95 °C for 10 min, followed by 40 cycles at 95 °C for 10 s, 60 °C for 1 min. Group specific standard curves were generated from serial dilutions of a known concentration of PCR products. Additionally, melting curve analysis was performed at the end of qPCR cycles to check the amplification specificity. Average values of the triplicate were used for enumerations of tested gene copy numbers for each group using standard curves generated 55. For all assays PCR efficiency was maintained above 90% with a correlation coefficient >0.99. Variations in copy number of targeted bacterial genera and *frc*- and *oxc*-genes were assessed using Mann-Whitney U test.

### Other analyses on 16S amplicon data

Effect of hyperoxaluric condition on microbial interactions were assessed using network analysis of co-occurrence and co-exclusion as described before^29^. In addition, the metabolic capabilities of bacterial community were inferred by utilizing a computational approach: PICRUSt (phylogenetic investigation of communities by reconstruction of unobserved states)^56^. Briefly, reference based OTU picking was performed in QIIME and the OTU table was imported to online PICRUSt tool at http://huttenhower.sph.harvard.edu/galaxy/ and functional predictions were made using KEGG Orthology (KO) database. The resulting data was analyzed using STAMP - version 2.0.2^57^. Functional gene predictions data was used for identification of oxalate bioconversion pathways present in gut microbiome.

## Additional Information

**How to cite this article**: Suryavanshi, M. V. *et al*. Hyperoxaluria leads to dysbiosis and drives selective enrichment of oxalate metabolizing bacterial species in recurrent kidney stone endures. *Sci. Rep.* **6**, 34712; doi: 10.1038/srep34712 (2016).

## Supplementary Material

Supplementary Information

Supplementary Table S3

Supplementary Table S5

## Figures and Tables

**Table 1:** Criteria for the grouping of the subjects.

Groups	KSD_FH	KSD_OX_COL	KSD_OX_N_COL	KSD_THIRD	HLT
Characteristics	KSD with family history	KSD with *Oxalobacter* colonization	KSD without *Oxalobacter* colonization	KSD with third episode	Healthy control
Samples Included	KSD3	KSD7	KSD12	KSD1	HLT8
	KSD9	KSD10	KSD20	KSD13	HLT9
	KSD21	KSD22	KSD29	KSD27	HLT10
	KSD25	KSD28	KSD31	KSD32	HLT11

**Table 2:** Table showing the targeted bacterial taxa, bacterial genes and their primers used for qPCR assays.

Target bacteria	Primers used	Sequence (5′-3′)	Amplicon size (bp)	Comments
Total bacteria^45^	341F	CCTACGGGAGGCAGCAG	177	For amplicon library generation and qPCR assay
	518R	ATTACCGCGGCTGCTGG		
Phylum Firmicutes^58^	FirmiF	CTGATGGAGCAACGCCGCGT	429	qPCR assay
	FirmiR	ACACYTAGYACTCATCGTTT		
Phylum Bacteroidetes^58^	BacterioF	CCGGAWTYATTGGGTTTAAAGGG	414	qPCR assay
	BacterioF	GGTAAGGTTCCTCGCGTA		
Genus *Lactobacillus* group^59^	F_Lacto 05	AGCAGTAGGGAATCTTCCA	352	qPCR assay
	R_Lacto 04	CGCCACTGGTGTTCYTCCATATA		
Genus *Bifidobacterium* group^60^	Bif16S3	AGGGTTCGATTCTGGCTCAG	156	qPCR assay
	Bif16S4	CATCCGGCATTACCACCC		
Genus *Prevotella* group^61^	PrevF	CACCAAGGCGACGATCA	283	qPCR assay
	PrevR	GGATAACGCCYGGACCT		
Genus *Feacalibacterium* group^46^	FPF	GGAGGAAGAAGGTCTTGCG	252	qPCR assay
	FPR	AATTCCGCCTACCTCTGCACT		
Genus *Megasphaera* group^46^	MegaF	CTAGTGGCAAACGGGTGAGT	179	qPCR assay
	MegaR	CAGACCGGCTACTGATCGTC		
Species *Oxalobacter formigenes (oxc-*gene)^62^	F_Oform oxc	CGACAATGTAGAGTTGACTGA	164	qPCR assay
	R_Oform oxc	CGTGTTGTTCGTGACGGAA		
Oxalotrophic bacteria (*frc-*gene)^15^	frc171_F	CTSTAYTTCACSATGCTSAAC	135	For amplicon library generation and qPCR assay
	frc306_R	GDSAAGCCCATVCGRTC		
