# Supplementary material for: Hyperoxaluria leads to dysbiosis and drives selective enrichment of oxalate metabolizing bacterial species in recurrent kidney stone endures
Source: Sci Rep. 2016 Oct 6;6:34712. doi: 10.1038/srep34712 (PMC5052600; doi:10.1038/srep34712)
Supplement: Supplementary Information [file srep34712-s1.pdf]

# Hyperoxaluria leads to dysbiosis and drives selective enrichment of oxalate metabolizing bacterial species in recurrent kidney stone endures

Mangesh V. Suryavanshi[#][a], Shrikant S. Bhute[#][b], Swapnil D. Jadhav[c], Manish S. Bhatia[c], Rahul P. Gune[d], Yogesh S. Shouche *[a]

**Supplementary Table S1:** Characteristics of recruited study subjects[1]

| Sample ID | Stone_ Episode | Position_Stone | Number_ stones | Family_ History | Diet | Age | Sex | Total_volume _Urine | SppGravity _urine | Oxalobacter_ Colonization | Urine_Oxalate |
|---|---|---|---|---|---|---|---|---|---|---|---|
| KSD1 | Third | Lower_Urinarytract | 7 | No | Occ_Nonveg | 32 | Male | 1500 | 1.01 | No | 140.6024 |
| KSD2 | Second | Upper_Urinarytract | 5 | No | Veg | 32 | Male | 2000 | 1.01 | No | 70.9542 |
| KSD3 | Second | Upper_Urinarytract | 5 | Yes | Occ_Nonveg | 34 | Male | 1200 | 1 | No | 40.0254 |
| KSD4 | Second | Upper_Urinarytract | 4 | No | Veg | 50 | Male | 1700 | 1.01 | No | 60.1502 |
| KSD7 | Second | Upper_Urinarytract | 5 | No | Occ_Nonveg | 29 | Male | 2000 | 1.005 | Yes | 80.8047 |
| KSD9 | Second | Lower_Urinarytract | 6 | Yes | Occ_Nonveg | 32 | Male | 1800 | Non_Reported | No | 100.6679 |
| KSD10 | Second | Upper_Urinarytract | 5 | No | Occ_Nonveg | 25 | Male | 1800 | Non_Reported | Yes | 70.4897 |
| KSD12 | Second | Upper_Urinarytract | 4 | No | Occ_Nonveg | 31 | Male | 2000 | Non_Reported | No | 50.9875 |
| KSD13 | Third | Upper_Urinarytract | 7 | No | Occ_Nonveg | 36 | Male | 2000 | Non_Reported | No | 130.8206 |
| KSD14 | Second | Upper_Urinarytract | 5 | No | Veg | 43 | Male | 2000 | Non_Reported | No | 90.5782 |
| KSD20 | Second | Upper_Urinarytract | 5 | No | Veg | 37 | Male | 1800 | 1.05 | No | 80.2496 |
| KSD21 | Second | Upper_Urinarytract | 8 | Yes | Occ_Nonveg | 50 | Male | 1800 | 1.01 | No | 150.2171 |
| KSD22 | Second | Lower_Urinarytract | 4 | No | Occ_Nonveg | 32 | Male | 1800 | 1.01 | Yes | 50.6745 |
| KSD23 | Second | Upper_Urinarytract | 4 | No | Occ_Nonveg | 28 | Male | 1800 | 1.01 | No | 50.8762 |
| KSD24 | Second | Upper_Urinarytract | 5 | No | Occ_Nonveg | 29 | Male | 1800 | 1.01 | No | 70.9864 |
| KSD25 | Second | Upper_Urinarytract | 6 | Yes | Occ_Nonveg | 50 | Male | 1800 | 1.01 | No | 120.9987 |
| KSD26 | Second | Upper_Urinarytract | 4 | No | Occ_Nonveg | 22 | Male | 1800 | 1.01 | No | 60.3574 |
| KSD27 | Third | Upper_Urinarytract | 5 | No | Veg | 37 | Male | 1800 | 1.01 | No | 70.6348 |
| KSD28 | Second | Upper_Urinarytract | 4 | No | Occ_Nonveg | 28 | Male | 1800 | 1.01 | Yes | 60.0269 |
| KSD29 | Second | Upper_Urinarytract | 3 | No | Occ_Nonveg | 43 | Male | 1800 | 1.01 | No | 50.3985 |
| KSD30 | Second | Upper_Urinarytract | 4 | No | Veg | 50 | Male | 1800 | Non_Reported | No | 50.4328 |
| KSD31 | Second | Upper_Urinarytract | 6 | No | Occ_Nonveg | 45 | Male | 2000 | 1.05 | No | 120.2586 |
| KSD32 | Third | Upper_Urinarytract | 5 | No | Occ_Nonveg | 41 | Male | 1500 | 1 | No | 60.9981 |
| KSD33 | Second | Upper_Urinarytract | 4 | No | Occ_Nonveg | 43 | Male | 2000 | Non_Reported | No | 50.6925 |

Continued on next page

| Sample ID | Stone_ Episode | Position_Stone | Number _stones | Family_ History | Diet | Age | Sex | Total_volume_ Urine | SppGravity_urine | Oxalobacter_ Colonization | Urine_Oxalate |
|---|---|---|---|---|---|---|---|---|---|---|---|
| HLT1 | No | NA | 0 | No | Occ_Nonveg | 38 | Male | 2000 | 1.015 | Yes | 2.0875 |
| HLT2 | No | NA | 0 | No | Occ_Nonveg | 42 | Male | 2000 | Non_Reported | Yes | 3.8035 |
| HLT3 | No | NA | 0 | No | Occ_Nonveg | 52 | Male | 2000 | Non_Reported | Yes | 0.5784 |
| HLT4 | No | NA | 0 | No | Occ_Nonveg | 25 | Male | 2000 | Non_Reported | Yes | 2.7721 |
| HLT5 | No | NA | 0 | No | Occ_Nonveg | 32 | Male | 2000 | Non_Reported | Yes | 2.1589 |
| HLT6 | No | NA | 0 | No | Veg | 36 | Male | 2000 | 1.01 | Yes | 1.9029 |
| HLT7 | No | NA | 0 | No | Veg | 45 | Male | 2000 | Non_Reported | Yes | 0.8565 |
| HLT8 | No | NA | 0 | No | Occ_Nonveg | 37 | Male | 2000 | Non_Reported | Yes | 11.4752 |
| HLT9 | No | NA | 0 | No | Occ_Nonveg | 51 | Male | 2000 | Non_Reported | Yes | 0.8812 |
| HLT10 | No | NA | 0 | No | Occ_Nonveg | 32 | Male | 1800 | Non_Reported | Yes | 0.4785 |
| HLT11 | No | NA | 0 | No | Occ_Nonveg | 28 | Male | 2000 | Non_Reported | Yes | 10.1217 |
| HLT12 | No | NA | 0 | No | Occ_Nonveg | 29 | Male | 1700 | 1 | Yes | 6.7485 |
| HLT13 | No | NA | 0 | No | Occ_Nonveg | 40 | Male | 2000 | 1.01 | Yes | 0.8979 |
| HLT14 | No | NA | 0 | No | Occ_Nonveg | 22 | Male | 2000 | 1.01 | Yes | 3.2486 |
| HLT15 | No | NA | 0 | No | Occ_Nonveg | 27 | Male | 2000 | 1.01 | Yes | 0.0573 |

**Supplementary Table S2:** Measures of bacterial alpha diversity indices

| Sample ID | chao1 | observed_species | PD_whole_tree | shannon | simpson |
|---|---|---|---|---|---|
| HLT1 | 3188.062 | 1568 | 3.33951 | 6.75439 | 0.942825 |
| HLT10 | 4147.134 | 2280 | 39.67672 | 7.900188 | 0.98444 |
| HLT11 | 4778.521 | 2483 | 44.84434 | 7.698131 | 0.975879 |
| HLT12 | 4531.065 | 2199 | 54.65652 | 9.037638 | 0.992895 |
| HLT13 | 2961.687 | 1311 | 36.08513 | 8.307108 | 0.987532 |
| HLT14 | 3952.469 | 2154 | 37.99856 | 7.809421 | 0.974925 |
| HLT15 | 6532.594 | 3793 | 59.04995 | 7.726033 | 0.968654 |
| HLT2 | 4035.278 | 1760 | 2.79373 | 7.78012 | 0.975384 |
| HLT3 | 4352.769 | 2235 | 2.95916 | 7.825879 | 0.975917 |
| HLT4 | 4573.644 | 2520 | 44.61908 | 8.113472 | 0.983384 |
| HLT5 | 4370.521 | 2287 | 2.78213 | 7.792071 | 0.977616 |
| HLT6 | 4547.443 | 2045 | 2.95584 | 7.872849 | 0.97791 |
| HLT7 | 1715.532 | 800 | 18.29441 | 6.966958 | 0.963885 |
| HLT8 | 2109.606 | 1093 | 23.45008 | 6.33274 | 0.936159 |
| HLT9 | 4877.569 | 2776 | 38.93904 | 7.711324 | 0.974653 |
| KSD1 | 3466.899 | 1710 | 1.8255 | 8.440399 | 0.991801 |
| KSD10 | 3558.939 | 1871 | 2.89247 | 7.906716 | 0.983112 |
| KSD12 | 3705.23 | 2018 | 2.91835 | 6.722392 | 0.957244 |
| KSD13 | 2828.123 | 1583 | 1.80824 | 7.361253 | 0.977771 |
| KSD14 | 3627.026 | 2119 | 1.38098 | 7.308434 | 0.973818 |
| KSD2 | 2274.427 | 1275 | 3.33582 | 6.776981 | 0.969376 |
| KSD20 | 2189.667 | 1177 | 2.452 | 7.086113 | 0.972638 |
| KSD21 | 2199.005 | 1299 | 2.6965 | 6.922358 | 0.97344 |
| KSD22 | 10305.73 | 6742 | 66.61992 | 7.422103 | 0.980141 |
| KSD23 | 2694.566 | 1524 | 1.09007 | 6.754543 | 0.948399 |
| KSD24 | 2549.641 | 1363 | 2.1313 | 6.901563 | 0.973064 |
| KSD25 | 4126.224 | 2169 | 3.079 | 7.540329 | 0.973658 |
| KSD26 | 6452.167 | 3453 | 5.18689 | 8.965894 | 0.991705 |
| KSD27 | 5248.79 | 2767 | 4.10664 | 8.537168 | 0.989064 |
| KSD28 | 6178.975 | 3377 | 4.27952 | 9.149567 | 0.993983 |
| KSD29 | 7144.502 | 3683 | 4.16099 | 8.904746 | 0.99108 |
| KSD3 | 3337.262 | 1736 | 2.37196 | 6.032698 | 0.922931 |
| KSD30 | 2773.22 | 1643 | 2.442 | 7.704705 | 0.98504 |
| KSD31 | 3785.719 | 1976 | 3.50901 | 7.405863 | 0.966777 |
| KSD32 | 3996.134 | 1979 | 2.40117 | 7.540159 | 0.97393 |
| KSD33 | 3410.762 | 1989 | 5.7882 | 8.058871 | 0.985998 |
| KSD4 | 3599.52 | 2258 | 2.48511 | 6.991653 | 0.964532 |
| KSD7 | 4309.941 | 2357 | 2.49431 | 7.46889 | 0.977564 |
| KSD9 | 3111.195 | 1593 | 3.49102 | 6.174241 | 0.930119 |

**Supplementary Table S4:** List of discriminant OTUs in HLT and KSD subjects as revealed by Random forest test

| OTU | HLT_mean | KSD_mean | taxonomy |
|---|---|---|---|
| denovo9141 | 607 | 1631.916667 | k__Bacteria;p__Bacteroidetes;c__Bacteroidia;o__Bacteroidales |
| denovo15755 | 1.266666667 | 264.3333333 | k__Bacteria;p__Bacteroidetes;c__Bacteroidia;o__Bacteroidales;f__Bacteroidaceae;g__Bacteroides;s__ |
| denovo12203 | 1.533333333 | 146.5833333 | k__Bacteria;p__Actinobacteria;c__Actinobacteria |
| denovo24792 | 23.46666667 | 106.2083333 | k__Bacteria;p__Firmicutes;c__Clostridia;o__Clostridiales;f__Lachnospiraceae |
| denovo1467 | 6.266666667 | 77.29166667 | k__Bacteria;p__Firmicutes;c__Clostridia;o__Clostridiales;f__Lachnospiraceae |
| denovo13645 | 0 | 67.875 | k__Bacteria;p__Actinobacteria;c__Actinobacteria |
| denovo10234 | 0.266666667 | 61.41666667 | k__Bacteria;p__Bacteroidetes;c__Bacteroidia;o__Bacteroidales;f__Bacteroidaceae;g__Bacteroides |
| denovo22044 | 5.066666667 | 58.95833333 | k__Bacteria;p__Firmicutes;c__Clostridia;o__Clostridiales;f__Lachnospiraceae |
| denovo8927 | 5.666666667 | 48.70833333 | k__Bacteria;p__Firmicutes;c__Clostridia;o__Clostridiales;f__Lachnospiraceae |
| denovo23675 | 4.133333333 | 27.95833333 | k__Bacteria;p__Firmicutes;c__Clostridia;o__Clostridiales;f__Lachnospiraceae |
| denovo27457 | 0.066666667 | 20.83333333 | k__Bacteria;p__Proteobacteria;c__Gammaproteobacteria |
| denovo10635 | 2.133333333 | 14.83333333 | k__Bacteria;p__Firmicutes;c__Clostridia;o__Clostridiales |
| denovo17711 | 0.933333333 | 10.45833333 | k__Bacteria;p__Firmicutes;c__Clostridia;o__Clostridiales;f__Lachnospiraceae |
| 330458 | 131.5333333 | 5.416666667 | k__Bacteria;p__Firmicutes;c__Clostridia;o__Clostridiales;f__Lachnospiraceae |
| denovo2817 | 0.133333333 | 4.375 | k__Bacteria;p__Firmicutes;c__Clostridia;o__Clostridiales;f__Lachnospiraceae |
| 178860 | 651.7333333 | 1.708333333 | k__Bacteria;p__Bacteroidetes;c__Bacteroidia;o__Bacteroidales;f__Prevotellaceae;g__Prevotella;s__copri |
| denovo25936 | 44.93333333 | 1.041666667 | k__Bacteria;p__Bacteroidetes;c__Bacteroidia;o__Bacteroidales;f__Prevotellaceae;g__Prevotella |
| 185411 | 24 | 0.916666667 | k__Bacteria;p__Firmicutes;c__Clostridia;o__Clostridiales;f__Lachnospiraceae |
| denovo24701 | 3.466666667 | 0.875 | k__Bacteria;p__Firmicutes;c__Clostridia;o__Clostridiales;f__Lachnospiraceae |
| denovo6254 | 112.7333333 | 0.25 | k__Bacteria;p__Bacteroidetes;c__Bacteroidia;o__Bacteroidales;f__Prevotellaceae;g__Prevotella;s__copri |
| 4456491 | 116.0666667 | 0.208333333 | k__Bacteria;p__Bacteroidetes;c__Bacteroidia;o__Bacteroidales;f__Prevotellaceae;g__Prevotella;s__copri |
| 364563 | 15.66666667 | 0.166666667 | k__Bacteria;p__Firmicutes;c__Clostridia;o__Clostridiales;f__Ruminococcaceae;g__Faecalibacterium;s__prausnitzii |
| 299382 | 29.6 | 0.125 | k__Bacteria;p__Bacteroidetes;c__Bacteroidia;o__Bacteroidales;f__Prevotellaceae;g__Prevotella |
| denovo3826 | 199.5333333 | 0.125 | k__Bacteria;p__Bacteroidetes;c__Bacteroidia;o__Bacteroidales;f__Prevotellaceae;g__Prevotella;s__copri |
| 363477 | 29.6 | 0.125 | k__Bacteria;p__Firmicutes;c__Clostridia;o__Clostridiales;f__Ruminococcaceae;g__Faecalibacterium;s__prausnitzii |
| 357002 | 13.13333333 | 0.083333333 | k__Bacteria;p__Firmicutes;c__Clostridia;o__Clostridiales;f__Lachnospiraceae |
| denovo18031 | 8.333333333 | 0.041666667 | k__Bacteria;p__Firmicutes;c__Clostridia;o__Clostridiales;f__Ruminococcaceae;g__Faecalibacterium;s__prausnitzii |
| denovo14074 | 7.666666667 | 0 | k__Bacteria;p__Bacteroidetes |
| 4307094 | 148.4 | 0 | k__Bacteria;p__Bacteroidetes;c__Bacteroidia;o__Bacteroidales |
| denovo20708 | 104 | 0 | k__Bacteria;p__Bacteroidetes;c__Bacteroidia;o__Bacteroidales;f__Prevotellaceae;g__Prevotella |
| denovo27485 | 15.66666667 | 0 | k__Bacteria;p__Bacteroidetes;c__Bacteroidia;o__Bacteroidales;f__Prevotellaceae;g__Prevotella |
| denovo6890 | 3.333333333 | 0 | k__Bacteria;p__Bacteroidetes;c__Bacteroidia;o__Bacteroidales;f__Prevotellaceae;g__Prevotella |
| denovo15129 | 3.333333333 | 0 | k__Bacteria;p__Bacteroidetes;c__Bacteroidia;o__Bacteroidales;f__Prevotellaceae;g__Prevotella |
| denovo6523 | 3.133333333 | 0 | k__Bacteria;p__Bacteroidetes;c__Bacteroidia;o__Bacteroidales;f__Prevotellaceae;g__Prevotella |
| denovo3669 | 2.466666667 | 0 | k__Bacteria;p__Bacteroidetes;c__Bacteroidia;o__Bacteroidales;f__Prevotellaceae;g__Prevotella |
| 346938 | 187.4666667 | 0 | k__Bacteria;p__Bacteroidetes;c__Bacteroidia;o__Bacteroidales;f__Prevotellaceae;g__Prevotella;s__copri |
| 178387 | 65.8 | 0 | k__Bacteria;p__Bacteroidetes;c__Bacteroidia;o__Bacteroidales;f__Prevotellaceae;g__Prevotella;s__copri |
| 292210 | 53.6 | 0 | k__Bacteria;p__Bacteroidetes;c__Bacteroidia;o__Bacteroidales;f__Prevotellaceae;g__Prevotella;s__copri |
| 509109 | 7.866666667 | 0 | k__Bacteria;p__Bacteroidetes;c__Bacteroidia;o__Bacteroidales;f__Prevotellaceae;g__Prevotella;s__copri |
| 301253 | 6.466666667 | 0 | k__Bacteria;p__Bacteroidetes;c__Bacteroidia;o__Bacteroidales;f__Prevotellaceae;g__Prevotella;s__copri |
| denovo10745 | 27.4 | 0 | k__Bacteria;p__Firmicutes;c__Clostridia;o__Clostridiales;f__Lachnospiraceae |
| 4425669 | 20.86666667 | 0 | k__Bacteria;p__Firmicutes;c__Clostridia;o__Clostridiales;f__Lachnospiraceae |
| 4341056 | 7.533333333 | 0 | k__Bacteria;p__Firmicutes;c__Clostridia;o__Clostridiales;f__Lachnospiraceae |
| 351200 | 4 | 0 | k__Bacteria;p__Firmicutes;c__Clostridia;o__Clostridiales;f__Lachnospiraceae |
| 363494 | 3.933333333 | 0 | k__Bacteria;p__Firmicutes;c__Clostridia;o__Clostridiales;f__Lachnospiraceae |
| 354563 | 1.733333333 | 0 | k__Bacteria;p__Firmicutes;c__Clostridia;o__Clostridiales;f__Lachnospiraceae |
| denovo1814 | 2.066666667 | 0 | k__Bacteria;p__Firmicutes;c__Clostridia;o__Clostridiales;f__Ruminococcaceae |
| denovo19660 | 1.666666667 | 0 | k__Bacteria;p__Firmicutes;c__Clostridia;o__Clostridiales;f__Ruminococcaceae;g__Faecalibacterium;s__prausnitzii |
| denovo12706 | 257.8666667 | 0 | k__Bacteria;p__Firmicutes;c__Clostridia;o__Clostridiales;f__Veillonellaceae;g__Dialister;s__ |
| denovo24505 | 78.93333333 | 0 | k__Bacteria;p__Firmicutes;c__Clostridia;o__Clostridiales;f__Veillonellaceae;g__Dialister;s__ |

**Supplementary Figure S6:** Detection Oxalobacter formigenes colonization using PCR: genus specific 16S rRNA gene amplification (416bp) (upper row) and oxc-gene amplification (318bp) (lower row) in representative subpopulation of HLT (annotated as H) and KSD (annotated as K) subjects

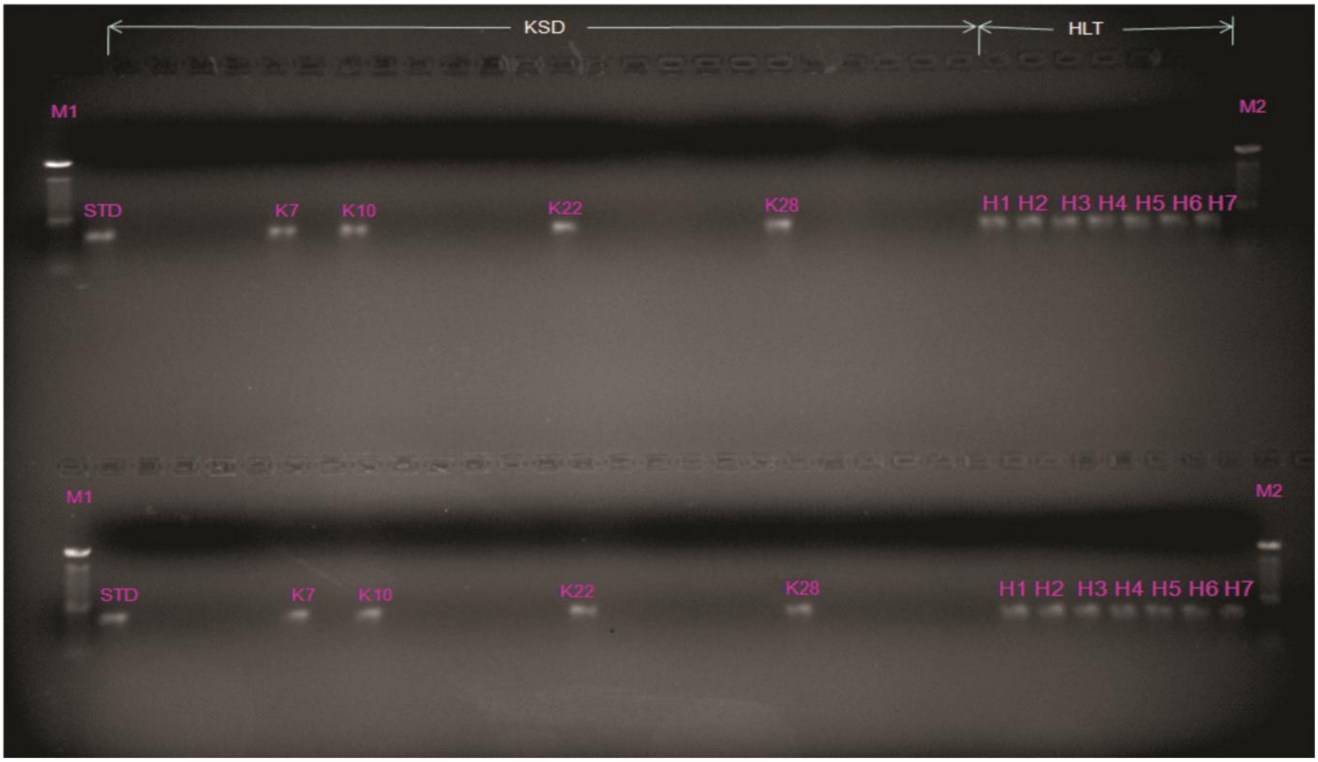

**Supplementary Figure S7:** *frc*-gene based PCR-DGGE fingerprint profile for representative subpopulation of HLT (red) and KSD (green) subjects

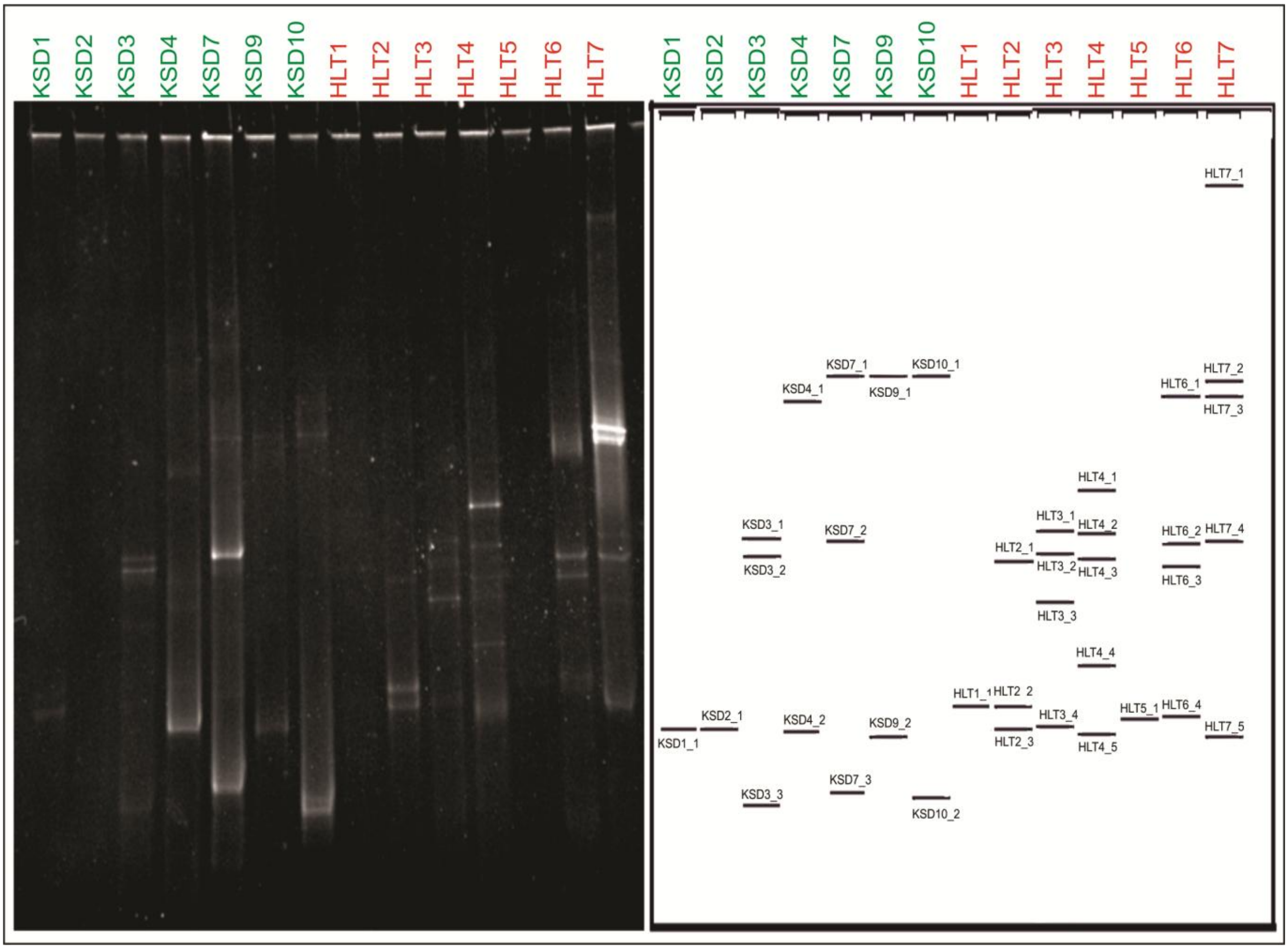

## Supplementary Methods S8

### Method for quantification of oxalate in 24 h urine sample[1]

**Preparation of standard solutions of oxalic acid**

Fifty mg of HPLC grade pure oxalic acid was weighted accurately. It was then transferred to 50 ml volumetric flask and volume was made up to the mark with pure methanol having concentration 1000 µg/ml. It was then sonicated and used for method development. After method optimization, solution of oxalic acid was prepared in mobile phase (Methanol: 0.001N Acetic Acid in Water (50:50, v/v)) of same strength (1000 µg/ml).

**Selection of wavelength of analysis**

The standard stock solution was diluted to10 µg/ml. The wavelength of analysis of oxalic acid was selected by analysing 10 µg/ml solution of same in pure methanol on UV-Visible Double Beam Spectrophotometer over 200 to 400 nm wavelength range. The wavelength of 237 nm was selected having maximum absorbance.

**Chromatographic method development for oxalate analysis in urine**

The sample solution of oxalic acid in urine for method development was prepared by mixing 0.1 ml of stock solution of oxalic acid in mobile phase and 2 ml of urine of HLT subjects and diluting it to 10 ml with mobile phase. This solution was then injected to chromatographic system with KYATECH HiQ Sil C18HS column using Rheodyne injector at flow rate of 1 mL/min and wavelength of analysis, 237 nm. The oxalic acid was resolved at 2.7 minute with good retention parameters under above chromatographic conditions. As concentration of oxalic acid was calculated by single point calibration method, same sample solution was then injected under similar conditions for 5 more times and area of oxalic acid peak was recorded using software. The mean of area of six readings of oxalic acid in urine was calculated and concentration of oxalic acid in urine was calculated using following formula,

Oxalic Acid content (µg/ml) = Area of oxalate peak / Mean of Area of 6 Readings × 10

The concentration of oxalic acid in mg / x ml of urine was calculated by multiplying above reading by dilution factor and x.

**Analysis of oxalate in quality control samples and in urine**

The quality control samples were prepared from standard stock solution and urine of HLT subjects and analyzed by same procedure given above. Similarly, analysis of urine of KSD and HLT subjects for oxalate content was carried out by method explained above without adding 0.1 ml of stock solution of oxalic acid in mobile phase and the results of analysis are reported.

### References


1. Suryavanshi, M. V. *et al.* HPLC analysis of human urine for oxalate content. *Int. J. Pharm. Pharm. Sci.* **8,** 13168; DOI: 10.22159//ijpps.2016.v8i12.13168 (2016).
